# The single-atom iron nanozyme mimicking peroxidase remodels energy metabolism and tumor immune landscape for synergistic chemodynamic therapy and photothermal therapy of triple-negative breast cancer

**DOI:** 10.3389/fbioe.2022.1026761

**Published:** 2022-10-26

**Authors:** Xiaojun Qian, Ronghua Shi, Jian Chen, Yong Wang, Xinghua Han, Yubei Sun, Cong Ling, Gang Wang, An-Wu Xu, Yueyin Pan

**Affiliations:** ^1^ Department of Medical Oncology, The First Affiliated Hospital of USTC, Division of Life Sciences and Medicine, University of Science and Technology of China, Hefei, China; ^2^ Core Facility Center for Life Sciences, School of Life Sciences, University of Science & Technology of China, Hefei, China; ^3^ Hefei National Laboratory for Physical Sciences at Microscale, University of Science and Technology of China, Hefei, China

**Keywords:** Fe-N-C single atom nanozymes, TNBC, CDT, PTT, energy metabolism, M-MDSC

## Abstract

Chemotherapy, as one main strategy to relieve tumor progression, has a weak effect on triple-negative breast cancer (TNBC) chest wall metastasis. The development of near-infrared (NIR) light-responsive nanomaterials for chemodynamic therapy (CDT) and photothermal therapy (PTT) is a promising platform but still challenging in biomedicine. This study reports a peroxidase mimicking nanozyme (Fe-N-C SAzyme) against TNBC by CDT and PTT. Fe-N-C SAzyme generated reactive oxygen species (ROS) by decomposing H_2_O_2_ into hydroxyl radicals (•OH) and also induced light-to-heat conversion under the exposure of 808 nm laser irradiation. With these biological characteristics, the obtained Fe-N-C SAzymes displayed enhanced cell cytotoxicity and inhibition of cancer cell proliferation both *in vitro* and *in vivo* at a low dose of nanoagent and a moderate NIR laser power density. Besides, Fe-N-C nanoagent with its excellent ROS generation brought metabolic reprogramming of elevated glycolysis in tumor cells. *In vivo* experiments, when combined with PTT, the enhanced antitumor effect was found by the elimination of M-MDSC in tumor microenvironment. Fe-N-C SAzymes can serve as a new synergistic CDT and PTT nanoagent to simultaneously reprogram tumor metabolism and tumor microenvironment. It will provide prospects for chemodynamic/photothermal combined cancer therapy for TNBC chest wall metastasis based on the use of a single nanosystem.

## Introduction

Triple-negative breast cancer (TNBC) chest wall recurrence is a common but vexing clinical problem for clinicians (Wakeam et al., 2018). Due to the ineffectiveness of tyrosine kinase inhibitors and endocrine therapy and the lack of local treatment, systemic medical treatments like chemotherapy, anti-angiogenesis therapy and immunotherapy are the only way to count (Arciero et al., 2018). With the development of nanomedicine, new strategies have emerged to make up the deficiencies of existing antitumor treatments. For example, nanoparticles deliver drug to tumors (Li et al., 2020) providing opportunities to enhance immunotherapy (Zhang and Pu, 2020) and induce cancer cells ferroptosis (Kim et al., 2016) in combination with chemodynamic therapy (CDT) and photothermal therapy (PTT) (Hu et al., 2021). Because of their enzyme-like activities, nanozymes have caused increasing interest in biosensing, biotherapeutics, and environmental science (Jiang et al., 2019). For the low-coordinated metal atoms often function as the catalytically active sites, single atom catalysts (SAzymes) with creating stable, finely dispersed metal clusters always possess a high catalytic activity and hold great prospects in photocatalysis, electrocatalysis and organocatalysis (Wei et al., 2020). Transition metal-N-doped carbon (M-N-C) can work as oxygen reduction reaction (ORR) in living systems (Wu and Zelenay, 2013). Among them, Fe atoms embedded nitrogen-doped carbon (Fe-N-C) nanomaterials show good dispersibility in water and biocompatibility, and could act as single atom nanozymes (SAzymes) to achieve artificial enzyme-catalyzed reactions. Through unraveling structure-property relationship, it is found that M-N-C SAzymes with dense metal atoms in peroxidase mimicking exhibit outstanding peroxidase-like activity (Jiao et al., 2020).

The excellent peroxidase-like catalytic performance of Fe-N-C SAzymes is closely associated with its strong capacity of adsorbing H_2_O_2_ molecules (high adsorption energy) and high capacity of •OH generation (low energy barrier). H_2_O_2_ plays a crucial part in biological tissues and is highly expressed in tumors. (Fu et al., 2018). The peroxidase mimicking nanozymes have been considered to catalyze the conversion of endogenous H_2_O_2_ to •OH that can enhance the phototoxicity of the encapsulated photosensitizers against hypoxic tumors (Jiao et al., 2020). Besides this CDT ability, PTT emerges based on the light-to-heat conversion due to their unique advantages of near-infrared (NIR) driven hyperthermia to kill cancer cells. The multifunctional therapy has been extensively developed as an active technique in the fight against cancer, due to its superior tissue penetration ability, high selectivity and low toxicity to normal tissues in contrast to chemotherapy and radiotherapy (Liu et al., 2018; Cheng et al., 2020; Wang et al., 2021). Multifunctional nanosystems are promising and effective antitumor drugs, showing unexpected biological functions like impact on apoptosis, (Odda et al., 2019), ferroptosis, (Huo et al., 2019), energy metabolism, (Zhou et al., 2014), and tumor immune microenvironment, (Liang et al., 2016), which have been proved to exhibit potential inhibitory effects against tumor growth and metastasis.

**FIGURE 1:**
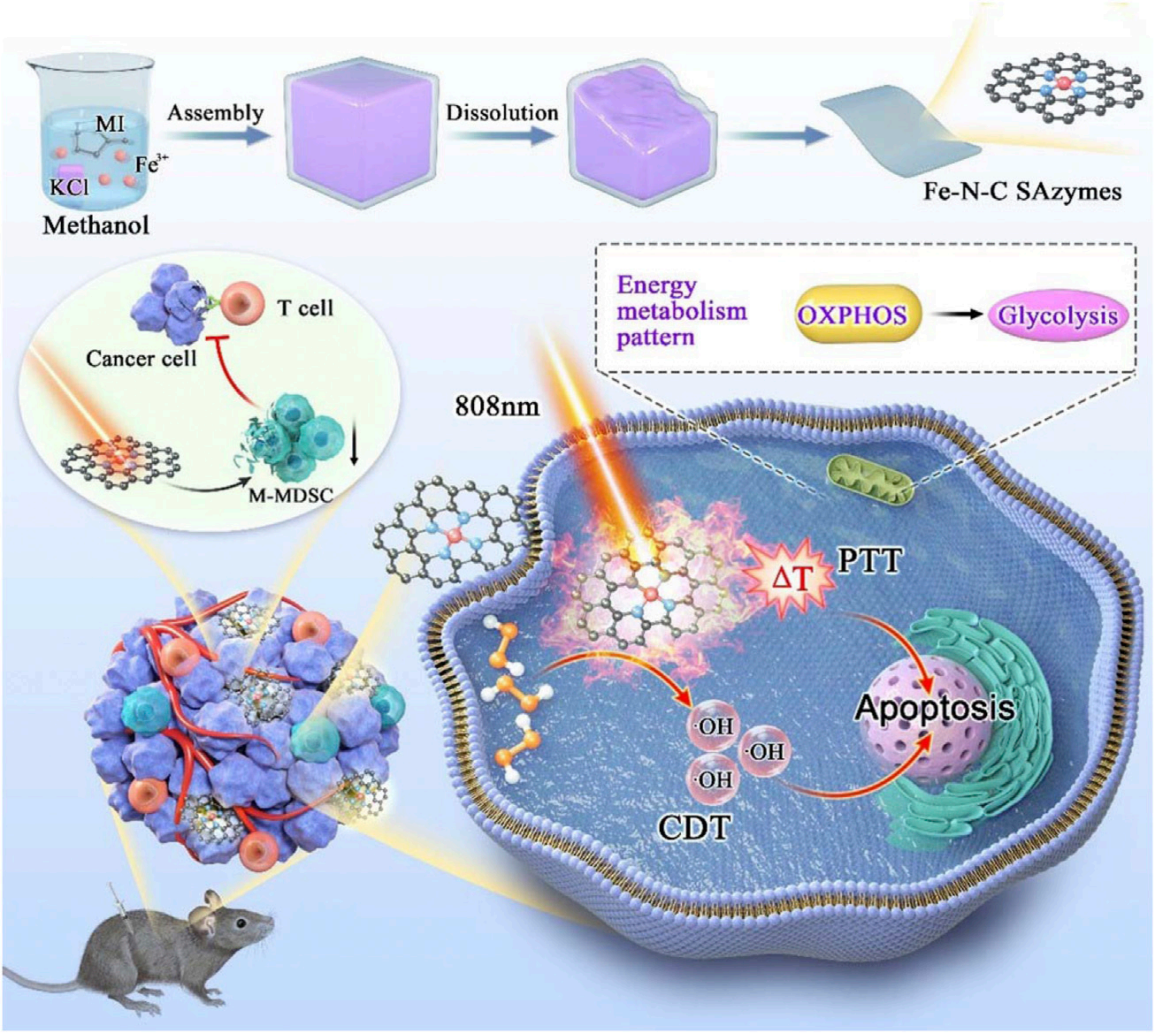
Schematic illustration of synthesis process of Fe-N-C SAzymes and the mechanism of cytotoxicity anticancer effect by remodeling energy metabolism and tumor immune landscape for synergistic CDT/PTT therapy.

Herein, we design and fabricate a novel Fe-N-C nanoagent, which displays outstanding peroxidase-like activity and unexpected antitumor function (Figure 1). This nanoagent utilize H_2_O_2_ in tumor cells and is subsequently completely oxidized to •OH to achieve tumor-specific targeting (Fu et al., 2018; Jiao et al., 2020). This oxidation process is independent of photothermia. Owing to the graphene thermal conductivity property, Fe-N-C nanoagent caused a moderate increase the temperature under a single-wavelength NIR laser which is more tolerant to body. Fe-N-C nanoagent which activate dual CDT/PTT treatment when irradiated with an 808 nm laser, is suitable for external laser radiation for chest wall metastasis. With the increased intracellular ROS, the biological effects of this peroxidase-like activity also alter the cellular energy metabolism pattern. In addition to tumor cells, tumor infiltrating immune cells are also affected by therapeutic methods. Fe-N-C SAzymes intratumoral injection combined with external laser irradiation caused immunosuppressive microenvironment destruction, which further inhibit the tumor growth.

## Materials and methods

### Chemicals and reagents

Iron (III) nitrate (Fe (NO_3_)_3_), dimethylimidazole (MI, C_5_H_8_N_2_), potassium chloride (KCl), were purchased from Shanghai Aladdin Biochemical Technology Co., Ltd. Indocyanine green (ICG) was purchased from Sagon Biotech. Methanol solution (CH_3_OH) and sulfuric acid (H_2_SO_4_ 98%) were acquired from Sinopharm Chemical Reagent Co., Ltd. Ferrostatin-1 (CAS#347174) was purchased from Selleck Chemicals. Anti-cleaved caspase-3 antibody (#9662) and anti-GPX4 (#52455) were purchased from Cell Signaling Technology. Anti-β-Actin antibody (20536-1-AP) was purchased from Proteintech. Goat anti mouse IgG-HRP (SC 2005) and Goat anti rabbit IgG-HRP (SC 2004) were purchased from Santa Cruz. Annexin V/7-AAG Apoptosis Detection Kit (C1062) was purchased from Beyotime Biotechnology. CCK-8 (BL001B) was purchased from Biomiky. CD45-V500 (662912), CD11b-PE-Cy7 (552850), CD4-APC-Cy7 (552051), CD8α-PerCP-Cy5.5 (551162), LY-6C-APC (560595) and LY-6G- PE (551461) were acquired from BD Pharmingen.

### Synthesis of Fe-N-C single-atom nanozymes

Fe-N-C SAzymes was synthesized by dissolving Ferric nitrate (2.5 mmol) and 2-methylimidazole (10 mmol) in 30 ml of methanol under stirring as reported in the literature (Jiao et al., 2020). Then, 500 g KCl was added with an intense stirring and the obtained reaction solution was heated at 80°C for 6 h in an oven. Obtained brown products were calcined under an argon atmosphere at 750°C (5°C/min) for 2 h. To remove the Fe nanoparticles and KCl template, the black product was ground into powder and washed once with 0.1 M H_2_SO_4_ solution and 3–4 times with ultrapure water. The product was dried in a vacuum oven at 85°C to obtain Fe-N-C SAzymes.

### Characterization

The morphology, structure and compositions of the nanomaterials were investigated by Scanning electron microscopy (SEM, JSM-6701F Japan), transmission electron microscopy (TEM, JEOL-2010 Japan) and high-angle annular dark-field scanning (HAADF-STEM, JEOL JEM-ARF200F Japan) at an accelerating voltage of 200 kV. Inductively coupled plasma optical emission spectrometer (ICP-OES) was carried out on the Agilent 720 ES. The crystal phase of the samples was analyzed by X-ray diffractometer (XRD, Rigaku, Japan) with Cu Kα radiation (*λ* = 0.154178 nm). DUV-3700 spectrophotometer (Shimadzu, Japan) was used to record the ultraviolet-visible-near-infrared (UV-Vis-NIR) optical absorption spectra ranging from 300 to 1,000 nm at room temperature.

### Photothermal conversion measurements

Fe-N-C nanoagent dispersed solutions with different concentrations (20, 50, 100, 500, and 1,000 μg/ml) were irradiated with laser doses of 0.8, 1.0, 1.5 and 1.8 W cm^−2^ at 808 nm (LSR808H, Ningbo Yuanming China). ICG solution and deionized (DI) water were also exposed to NIR laser under the same conditions. Temperature changes were monitored every 1 min by a digital thermometer for indicated time.

### Peroxidase-mimicking assays

Peroxidase-mimicking activity of the Fe-N-C SAzymes was tested out by •OH generation detection using TMB (Aladdin T100417) as the substrate with the addition of H_2_O_2_ in the HAc-NaAc buffer solution (pH 4.5) at room temperature. The mixed solution turned blue after 2 min and the absorbance at 652 nm was detected by using the UV-vis spectrophotometer. For a typical chromogenic reaction system to detect •OH generation and demonstrate POD-like activity, 80 μL of TMB (2 mM), 60 μL H_2_O_2_ (100 mM) and 20 μL of Fe-N-C (0.5 mg/ml) were added in Hac-NaAc buffer to a total volume of 2 ml. Intracellular •OH assay was performed by putting MDA-231 or 4T1 cells, TMB solution (160 μL, 2 mM) and Fe-N-C SAzymes (0.5 mg/ml) with or without GSH (162 mM) into Hac-NaAc buffer (pH 4.5) to a total of 2 ml. The mixture turned blue after incubation for 10 min and the absorbance of 652 nm was detected by UV-vis spectrophotometer. The color of the mixed solutions changed depending on the various amounts of catalyst in the chromogenic reaction system, which was indicated by the absorbance at 652 nm.

### Cell culture

Luciferase-labeled 4T1, MDA-MB-231 and MCF-10A cells were kindly obtained from Professor Rongbin Zhou, the School of Life Sciences, University of Science and Technology of China. Cells were cultured at 37°C and 5% CO_2_ in Dulbecco’s modified Eagle’s medium (DMEM) supplemented with 10% FBS.

### CCK-8 cell viability assay

Cells were seeded in 96-well plates at a density of 8,000–10,000 cells per well. For CDT in combination with PTT treatment, after Fe-N-C added for 4 h, the cells were irradiated with NIR laser (808 nm) for indicated time. After different treatments, 10 μL CCK-8 per well was added and incubated for 4 h at 37°C following the instructions. The absorbance was measured *via* spectrophotometer (Elx800, BioTek, Winooski, VT, United States ) at 450 nm.

### Apoptosis assay

4 × 10^4^ cells in 1.5 ml tube were treated by Fe-N-C or NIR laser (808 nm). Apoptosis assay was carried out by Annexin V/7-AAD reagent kit according to the manufacturer’s protocol. Briefly, after treatment, cells were washed twice by PBS, and then Annexin V-FITC stock solution (5 μL) and 7-AAD-PE (10 μL) were added and incubated for 5 min at room temperature in the dark. The samples were immediately analyzed by florescence-activated cell sorting (FACS Verse BD).

### Immunoblot

SDS loading buffer was added to treated cells, and then boiled for 10 min. Samples were separated by electrophoresis on a 15% sodium dodecyl sulfate (SDS)-polyacrylamide gel. Then the protein was transferred to the PVDF membrane. After blocked by TBST with 5% non-fat dry milk for 2 h, the PVDF membrane was incubated with primary antibodies at 4°C overnight, followed by HRP-conjugated secondary antibody for 1 h. Proteins were detected *via* molecular image (ChemiDoc™ XRS + BIO-RAD).

### Real-time quantitative PCR assay

Cells were seeded in 6-well plate and treated with Fe-N-C at indicated concentration for 4 h. RNA was isolated using the TRIzol reagent (Thermo Fisher) and reversed to cDNA using Superscript Vilo cDNA synthesis kit (Thermo Fisher). RT-qPCR assay was performed with SYBR premix ex Taq (Takara) using BioRad CXF96 Sequence Detection System (Applied Biosystems). The following primers for human genes were used (F: forward; R: reverse):

GPX4 F:5′- CCG​ATA​CGC​TGA​GTG​TGG​TT -3′,

R: 5′- AGC​CGT​TCT​TGT​CGA​TGA​GG -3′, 

PTGS2 F:5′- GGC​CAT​GGG​GTG​GAC​TTA​AA -3′,

R: 5′- CCC​CAC​AGC​AAA​CCG​TAG​AT -3′, 

FTH1 F:5′- AGC​TCT​ACG​CCT​CCT​ACG​TT -3′,

R: 5′- GTG​GCC​AGT​TTG​TGC​AGT​TC -3′, 

ACSL4 F:5′- TGC​TCA​CCA​TTA​TTT​TGC​TGC​C -3′,

R: 5′- GTC​GAA​GTG​TGT​GAC​AGA​GC -3′, 

GAPDH F: 5′- GTC​AAG​GCT​GAG​AAC​GGG​AA′, 

R:5′-AAATGAGCCCCAGCCTTCTC -3′.

### Mitochondrial OXPHOS and glycolysis rate assay

2 × 10^4^ cells per well were seeded in a Seahorse XF96 Cell Culture Microplate. Cells were treated with Fe-N-C for 4 h followed by washing twice with Agilent Seahorse XF medium. For mitochondrial OXPHOS (OCR) analysis, the medium contained 10 mM glucose solution, 1 mM pyruvate solution and 2 mM glutamine solution, while for proton efflux rate (PER) and extracellular acidification (ECAR) analysis, the medium contained 1 ml (10 mM) glucose solution, 1 mM pyruvate solution, 2 mM glutamine solution and 0.5% HEPS. Cells were then incubated in a CO_2_-free incubator for 1 h prior to measurement. Mitochondrial OXPHOS and glycolysis rate or stress were measured by Seahorse XF^e^96 extracellular flux analyzer (Agilent). During measurement, oligomycin (1.5 μM), carbonyl cyanide p-trifluoromethoxyphenylhydrazone (FCCP 1 μM), antimycin A and rotenone (0.5 μM) were added in order for OCR analysis. For PER analysis antimycin A and rotenone (0.5 μM) and 2-deoxyglucose (50 mM) were added in order, however, for ECAR analysis glucose (10 mM), oligomycin (1.5 μM) and 2-deoxyglucose (50 mM) were added. Each group contained at least three multiple wells.

### Mice tumor models

6–8 weeks old female B6 mice were purchased from SLAC Laboratory Animal Co., Ltd. (Shanghai, China). All animals received care in compliance with all relevant ethical regulations regarding animal research. The *in vivo* experiments were approved by The Ethics Committee of University of Science and Technology of China. To establish tumor model, 8 × 10^5^ luciferase-labeled 4T1 cells in 100 μL of PBS were subcutaneous injected. After 10 days, the volume of about 150 mm^3^ was observed. The tumor length, width, and body weight were measured every 3 days. 200 μg of Fe-N-C in 100 μL PBS was carried out intratumoral injection. After 24 h, the tumors were exposed to NIR laser (808 nm 1.0 W cm^−2^) for 10 min. Tumor surface temperature was recorded during irradiation. All mice were sacrificed after 21 days, and the tumors were stripped and weighed.

### Flow cytometry and cell sorting

The stripped mammary tumors were cut into small pieces and digested with collagenase Ⅳ (400 U ml^−1^ Sigma-Aldrich) and 3% FBS (Gibco) for 30 min in a shaking incubator (150 r.p.m., 37°C). The suspension containing tumor cells was filtered using a cell strainer (70 μm) and was centrifuged to obtain single-cell. Cells were stained with fluorochrome-conjugated antibodies and analyzed using FACS Verse (BD).

### Statistics and reproducibility

For tumor growth and temperature curves, two-way analysis of variance (ANOVA) was carried out to tell the differences between groups in the GraphPad Software. Other statistical analyses were made by two-sided unpaired Student’s *t*-tests in GraphPad Prism or Excel and are presented as mean ± S.E.M. *p* < 0.05, which was considered to be significant.

## Results

### Identification of Fe-N-C SAzymes

**FIGURE 2:**
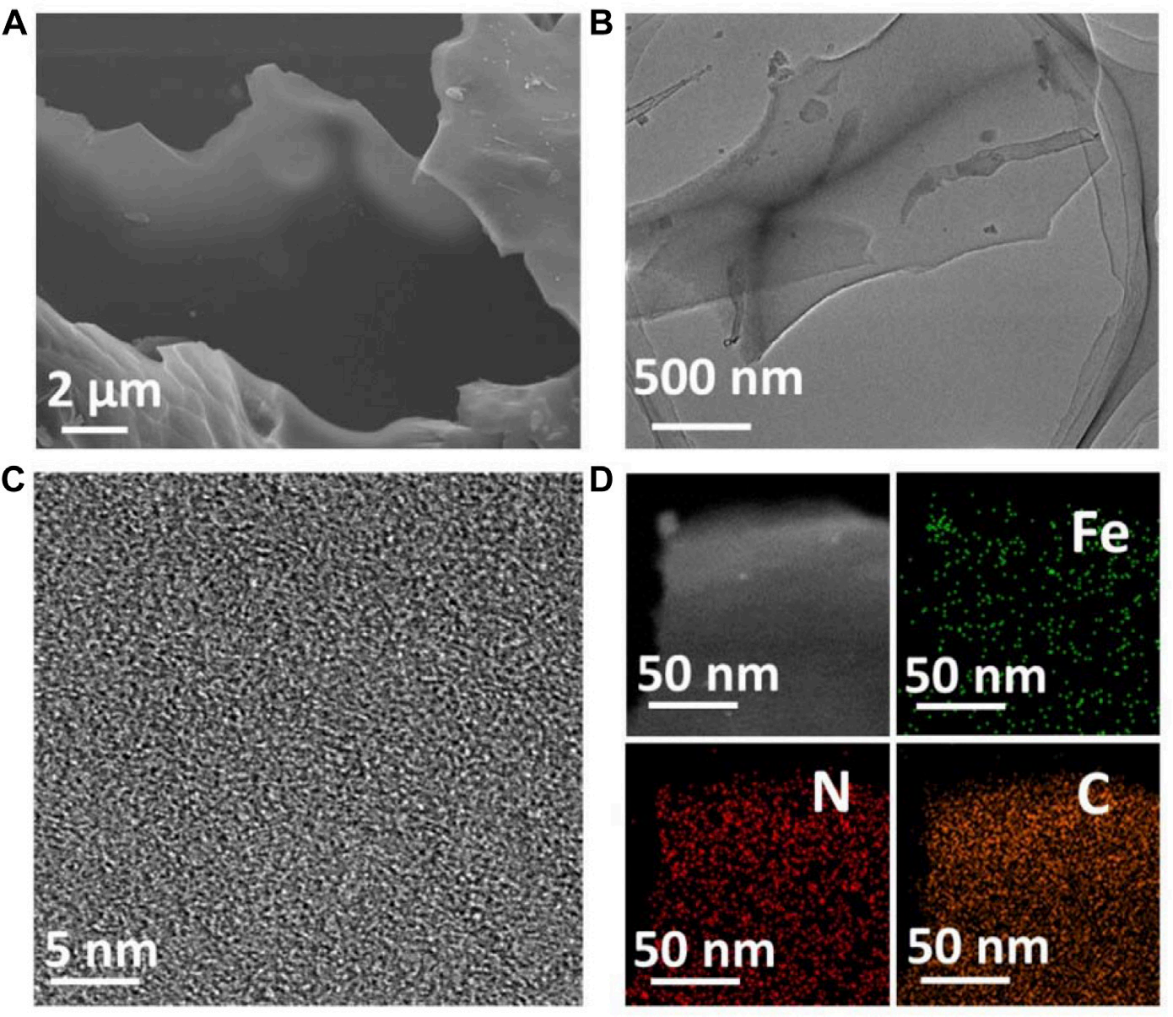
**(A)** SEM image of Fe-N-C SAzymes. **(B)** TEM image of Fe-N-C SAzymes. **(C)** HRTEM image of Fe-N-C SAzymes. **(D)** HADDF-STEM image and EDX elemental mapping images of Fe-N-C SAzymes.

Fe-N-C single-atom nanozymes (Fe-N-C SAzymes) were synthesized by pyrolysis of well-mixed iron (III) nitrate and dimethylimidazole (MI) in methanol solution with the template of KCl, then followed by acid etching according to a previously reported method (See the methods section) (Jiao et al., 2020). The resulting nanomaterials were characterized using scanning electron microscopy (SEM) and transmission electron microscope (TEM), which demonstrated that obtained Fe-N-C SAzymes possess ultrathin nanosheets with good dispersibility in water (Figures 2A,B). X-ray diffraction (XRD) pattern showed that peaks appeared at 23.8° and 42.9° were assigned to the (002) and (101) planes of graphitic carbon (Supplementary Figure S1). (Lin et al., 2014) It was noted that no Fe characteristic peaks were detected, indicating the absence of Fe nanoparticles, in agreement with HRTEM result (Figure 2C). The nanomaterials were then measured by high-angle annular dark-field scanning TEM (HADDF-STEM) and corresponding energy-dispersive X-ray (EDX) elemental analysis which showed the elemental carbon, nitrogen, and iron were evenly distributed in nanosheets (Figure 2D). Inductively coupled plasma optical emission spectroscopy (ICP-OES) analysis exhibited a high Fe single-atom loading of about 13.4 wt%. The distinct optical absorption properties of the as-made nanoagent were investigated by UV-vis-near-IR (UV-vis-NIR) spectra, and as shown in Supplementary Figure S2, Fe-N-C exhibited very broad optical absorption ranging from 200 to 1,000 nm, which means our sample can respond to various wavelengths of NIR.

### Photothermal conversion performance of Fe-N-C SAzymes

**FIGURE 3:**
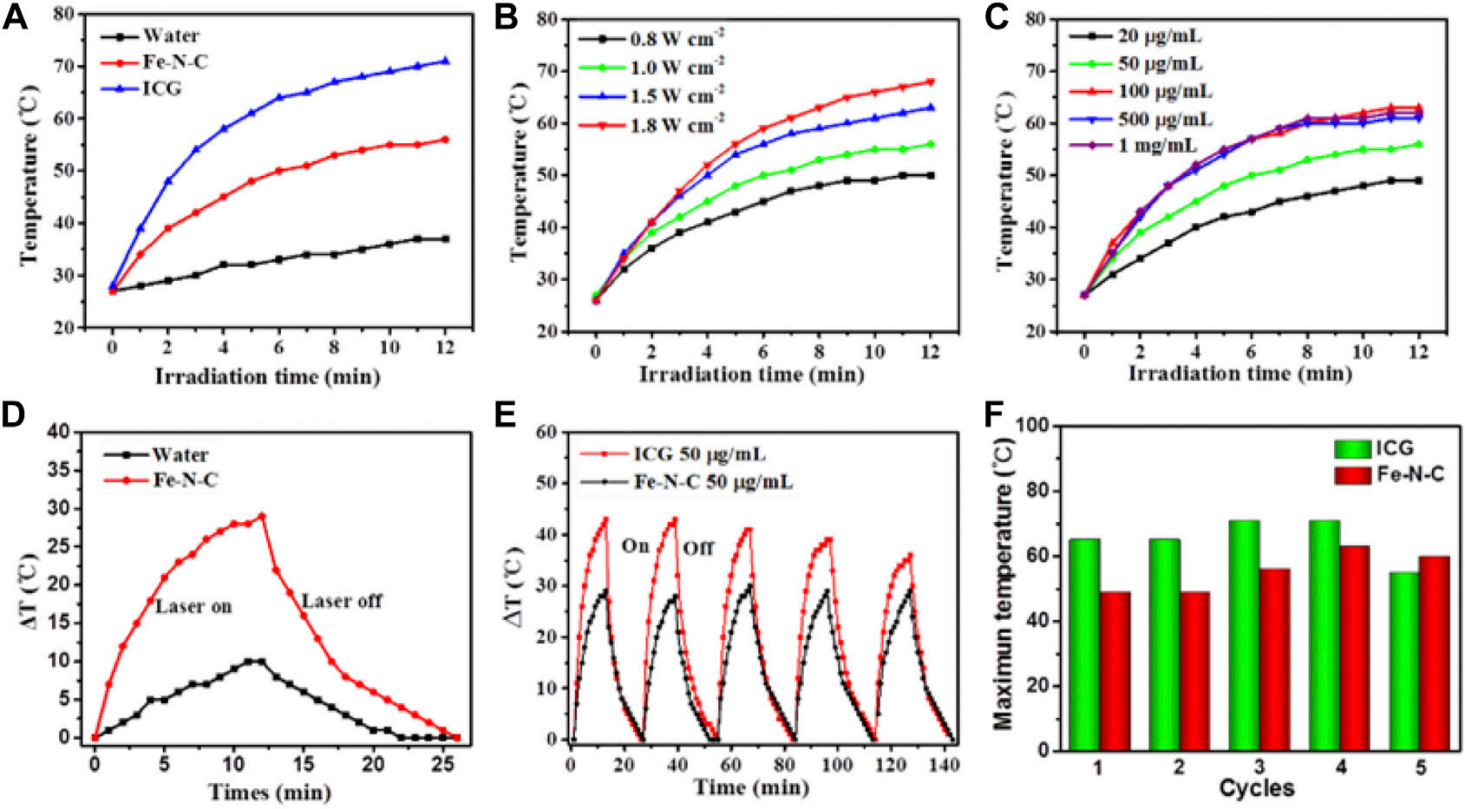
Photothermal conversion performance. **(A)** Fe-N-C SAzymes, ICG aqueous solutions and pure water under irradiation with an 808 nm laser at a power density of 1.0 W cm^−2^ for 12 min. **(B)** Fe-N-C SAzymes dispersion solutions with the constant concentration of 50 μg/ml under 808 nm laser irradiation at different power density of 0.8, 1.0, 1.5, 1.8 Wcm^−2^ for 12 min. **(C)** Fe-N-C SAzymes dispersion solutions with different concentrations (20, 50, 100, 500 and, 1,000 μg/ml) under 808 nm laser irradiation at a power density of 1.0 Wcm^−2^ for 12 min. **(D)** Heating and cooling curves of Fe-N-C SAzymes aqueous solution and pure water irradiated with an 808 nm NIR laser (1.0 W cm^−2^). **(E)** Stability of Fe-N-C SAzymes and ICG aqueous solutions under irradiation with an 808 nm laser at a power density of 1.0 W cm^−2^ for five on/off cycles. **(F)** The maximum temperature changes during five cycles between Fe-N-C SAzymes and ICG aqueous solutions after 808 nm laser irradiation. Data are representative of three independent experiments.

According to UV-vis-NIR result, the strong NIR absorption of the sample endows its moderate property of light-to-heat conversion within all wavelengths, therefore we selected 808 nm laser irradiation for further studies. To determine the photothermal performance of the Fe-N-C SAzymes dispersed aqueous solution under NIR light irradiation, various laser times, power densities, and concentrations were used. Fe-N-C SAzymes presented a mild temperature increase when irradiated with an 808 nm laser compared to Indocyanine green (ICG) (Figure 3A). ICG, as an FDA-approved photothermal agent raised the temperature from 28°C to 69°C within 10 min, while Fe-N-C nanoagent only caused the temperature increase from 28°C to 56°C under the same conditions, which was safer in clinical applications. Pure water was used as a control and showed a little increase in temperature less than 42°C (Figure 3A) which was considered as the minimum working temperature to kill tumor cells. Like other photothermal conversion nanomaterials, the temperature induced by Fe-N-C increased when the power density rose from 0.8 to 1.8 W cm^−2^ (Figure 3B). Another security characteristic was that when Fe-N-C concentrations increased, the temperature raised, but remained stable even when the concentration increased to 1,000 μg/ml (Figure 3C). When the laser was turned off, the temperature rapidly dropped to room temperature (Figure 3D). To compare the stability of the Fe-N-C and ICG, the temperature changes during the five cycles of laser turning on and off were examined. The result indicated that the maximum temperature of ICG decreased after five cycles but did not change in Fe-N-C, which meant it had higher photothermal stability than ICG (Figures 3E,F). These results proved the moderate and stable photothermal conversion capabilities of novel Fe-N-C SAzymes.

### The capacity of hydroxyl radical •OH generation by Fe-N-C SAzymes

**FIGURE 4:**
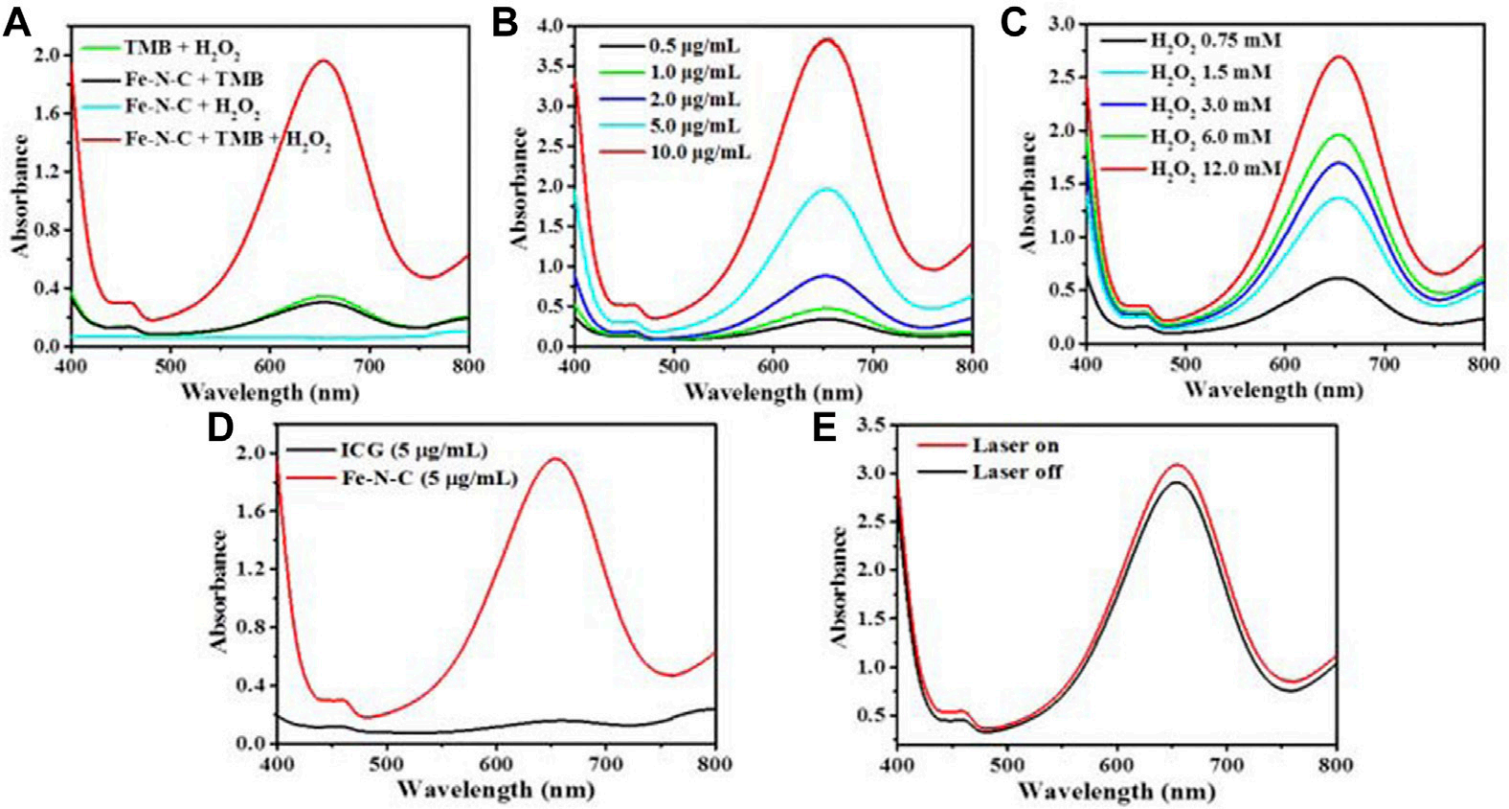
The capacity of •OH generation by Fe-N-C SAzymes. **(A)** Detection of peroxidase (POD)-like activity of Fe-N-C (5 μg/ml) through colorimetric assays of TMB as substrate (within 120 s) by UV-Vis-NIR absorption spectra at 652 nm. **(B)** UV-vis absorption spectra for the •OH generation by various concentrations of Fe-N-C at the same concentration of H_2_O_2_ (3 mM). **(C)** UV-vis absorption spectra for the generation of •OH by Fe-N-C (5 μg/ml) with various concentrations of H_2_O_2_. **(D)** Comparison of peroxidase (POD)-like activity of Fe-N-C and ICG through chromogenic assay of TMB as substrate (within 120 s) at the same concentration. **(E)** Peroxidase-like activity with or without NIR laser irradiation at the same concentration of Fe-N-C (5 μg/ml). Data are representative of two independent experiments.

Generally, Fe-N-C SAzymes possess Fe atoms as a coordination center followed by 4 N atoms as ligands, which obviously shows a similar coordination structure to natural peroxidase. So, the intrinsic enzyme-like activity of the obtained Fe-N-C SAzymes was investigated by typically chromogenic reaction at 652 nm using 3,3′,5,5′-tetramethylbenzidine (TMB) with the introduction of H_2_O_2_. Fe-N-C SAzymes exhibited good specific activity for catalyzing H_2_O_2_ conversion to hydroxyl radicals (•OH) (Figure 4A). The peroxidase-like activity was enhanced either when the Fe-N-C concentration or the H_2_O_2_ concentration increased (Figures 4B,C). As a phototherapy agent, ICG did not have the ability to generate •OH (Figure 4D). After 10 min of 808 nm laser irradiation, Fe-N-C did not cause an extra strong absorbance. It was noted that the intrinsic peroxidase activity of Fe-N-C did not require NIR (Figure 4E). These results proved that Fe-N-C exhibited an excellent peroxidase-like activity to convert H_2_O_2_ to •OH regardless of NIR irradiation.

### CDT/PTT *in vitro*

**FIGURE 5:**
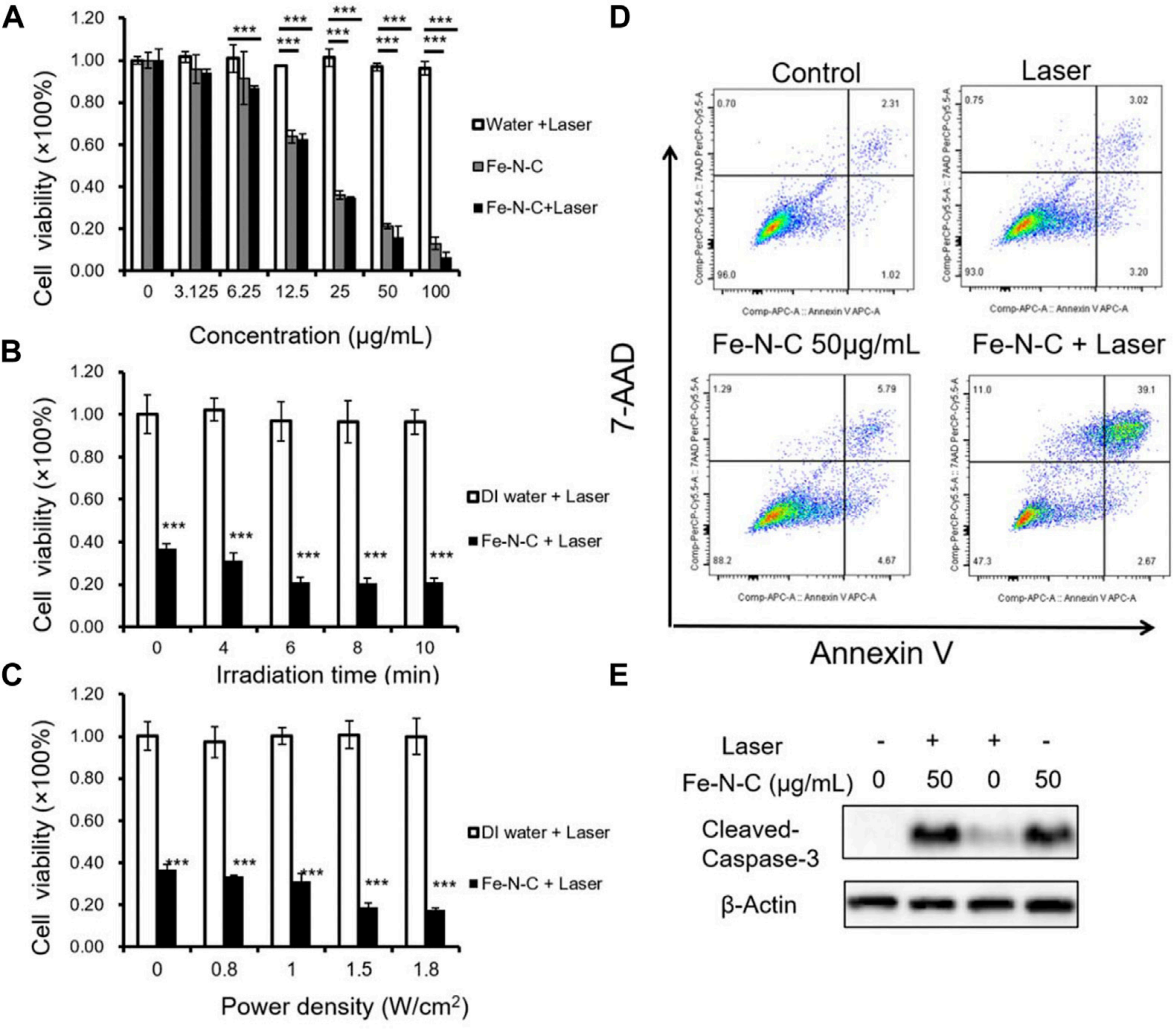
CDT/PTT *in vitro*. **(A)** MDA-MB-231 cells were treated by Fe-N-C with different concentrations for 4 h and then exposed to a 1.0 W cm^−2^ laser for 10 min. The cell viability was measured by CCK8 assay. **(B)** Cell viability of MDA-MB-231 cells treated with Fe-N-C (25 μg/ml) for 4 h and then exposed to a 1.0 W cm^−2^ laser for different times. **(C)** MDA-MB-231 cells were treated with Fe-N-C (25 μg/ml) for 4 h and then exposed to 808 nm laser of different power densities for 10 min to detect cell viability. **(D)** MDA-MB-231 cells were treated with 50 μg/ml of Fe-N-C for 4 h and then exposed to a 1.0 W cm^−2^ laser for 10 min or not, following testing with Annexin V/7-AAD assay. **(E)** MDA-MB-231 cells were treated with or without 50 μg/ml of Fe-N-C for 4 h and then exposed to a 1.0 W cm^−2^ laser for 10 min or not, following Western-Blot assay. Data are representative of three **(D,E)** or four **(A–C)** independent experiments. Summary data are shown as the mean ± S.E.M. with *p* values determined by two-tailed Student’s *t*-test (*p < 0.05, **p < 0.01, ***p < 0.001).

In contrast to normal cells, tumor cells accumulate a lot of H_2_O_2_, which enhances the adhesion of tumor cells and cause cell proliferation, division and metastasis. It is noted that tumor cells exhibit a strong dependence on H_2_O_2_ concentration, and are also very sensitive to its change. Tumor cells cannot tolerate excessive increase or decrease in H_2_O_2_ burden, therefore, H_2_O_2_ could serve as a potential biomarker. By converting H_2_O_2_ to •OH, Fe-N-C SAzymes not only downregulate the H_2_O_2_ concentration but also produce a large amount of •OH, which is a main member of ROS playing an important role in antitumor therapy. To explore the potential antitumor effect of Fe-N-C SAzymes, we next surveyed the antitumor activity in a human triple-negative breast cancer (TNBC) cell line MDAMB-231. TNBC is defined as a tumor lacking the expression of estrogen receptor, progesterone receptor and HER2, so there is no targeted therapy and hormone therapy available. Chest wall metastasis is common in TNBC, and chemotherapy is the main strategy to relieve tumor progression. Due to poor drug penetration, tumor may undergo ulceration and exudation that leads to inconvenient life and serious suffering. It is urgent to develop some new effective strategies to overcome this situation. CCK-8 cell viability assay showed that Fe-N-C SAzymes induced a dose-dependent decrease of cell viability and displayed enhanced antitumor activity at high-dose after 808 nm laser irradiation. Under 10 min irradiation, 100 μg/ml Fe-N-C SAzymes-mediated phototherapy achieved exceptional antitumor efficacy with >90% tumor growth inhibition, that the cell viability decreased from 13% to 6% (Figure 5A). Additionally, Fe-N-C SAzymes displayed time and power density dependent decrease of cell viability when a dose of 25 μg/ml was used (Figures 5B,C). To further verify, Annexin V/7-AAD apoptosis assay was carried out, and the results indicated that Fe-N-C treatment caused 5.79% of late apoptotic cells without laser illumination, and 39.1% after laser exposure (Figure 5D). The similar result was obtained by caspase-3 cleavage detection, another indicator for activation of apoptosis (Figure 5E). Taken together, these results proved that obtained Fe-N-C SAzymes exhibited highly effective CDT/PTT performance in a human TNBC cell line.

### Fe-N-C chemodynamic therapy activity caused by •OH generation

**FIGURE 6:**
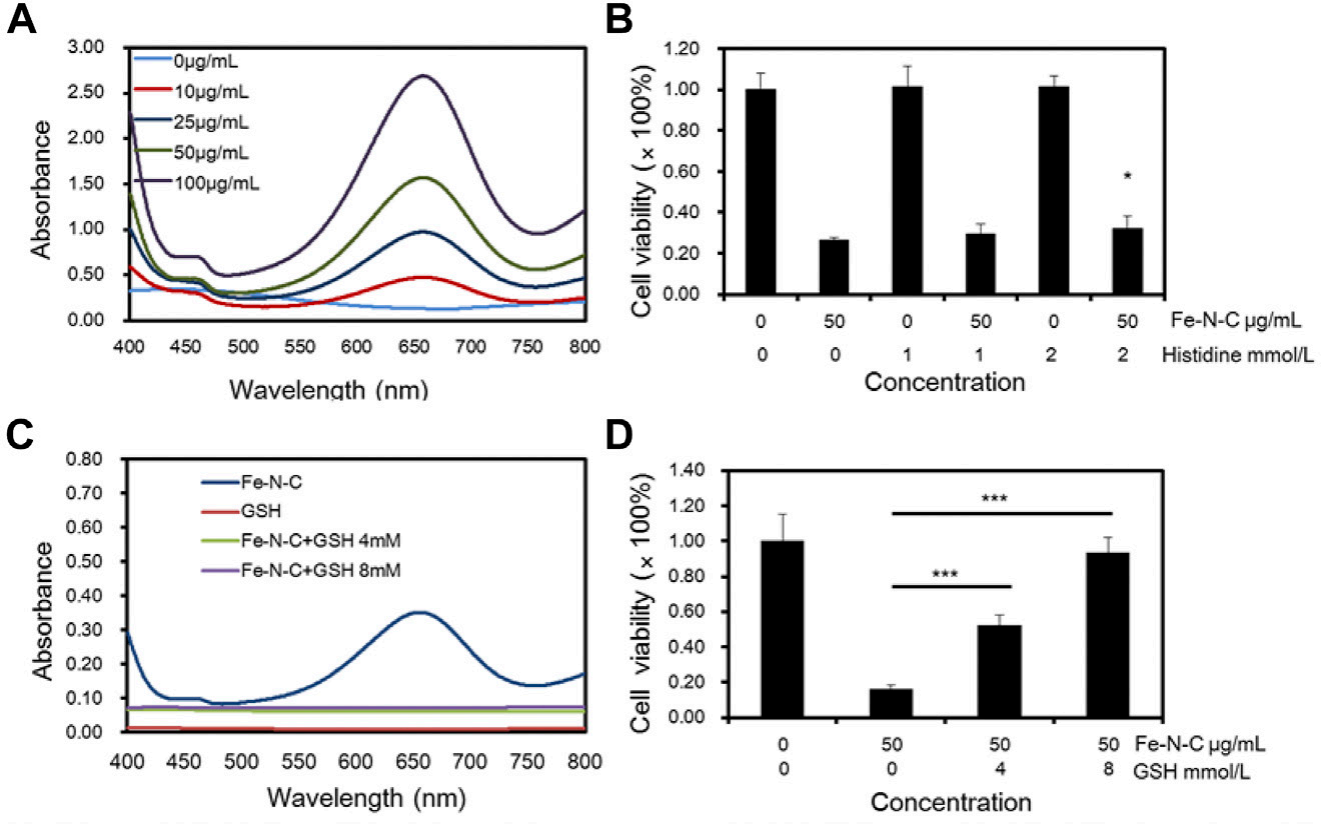
Fe-N-C CDT activity caused by •OH generation. **(A)** Intracellular H_2_O_2_ detection through colorimetric assay of TMB as substrate (within 10 min) by UV-Vis-NIR absorption spectra at 652 nm with different Fe-N-C concentration in MDA-MB-231 cells. **(B)** MDA-MB-231 cells viability was detected by CCK-8 assay, that cells were treated with histidine following by 50 μg/ml Fe-N-C for 4 h. **(C)** Intracellular H_2_O_2_ detection by UV-Vis-NIR absorption spectra with or without GSH addition. Each group contained 1 × 10^5^ MDA-MB-231 cells and Fe-N-C 25 μg/ml. **(D)** Cell viability of MDA-MB-231 cells treated with GSH following by Fe-N-C with 50 μg/ml for 4 h. Data are representative of three **(A,D)** or four **(B,C)** independent experiments. Summary data are shown as the mean ± S.E.M. with *p* values determined by two-tailed Student’s t-test (*p < 0.05, **p < 0.01, ***p < 0.001).

The main biological effect of Fe-N-C nanoagent is the conversion of H_2_O_2_ to •OH, and •OH is considered to be the main cause of antitumor activity. The •OH in TNBC cells was investigated by colorimetric assay with TMB as substrate. First, •OH was detected in normal human breast cell line MCF-10A and MDA-MB231 and found that •OH was elevated in tumor cells (Supplementary Figure S4). Then, gradually increasing drug concentrations were added and the results showed that Fe-N-C induced •OH generation increased in a dose-dependent manner (Figure 6A). To investigate the mechanism of Fe-N-C CDT activity, histidine and GSH were used to neutralize •OH. CCK-8 assay showed that after •OH was neutralized, cell viability increased dramatically (Figures 6B,D), while in the absence of histidine, Fe-N-C treatment caused a 73.7% increase in cancer cell death. GSH was considered to be a strong •OH scavenger, dramatically blocked intracellular •OH generation in MDA-MB-231 cells (Figure 6C). With the addition of GSH (8 mM), cell viability increased to 93.41% (Figure 6D). These results proved that Fe-N-C SAzymes generate •OH radicals to exhibit potent antitumor activity *in vitro*.

### Fe-N-C SAzymes elevate glycolysis to modulate tumor mitochondrial energy metabolism reprogramming

**FIGURE 7:**
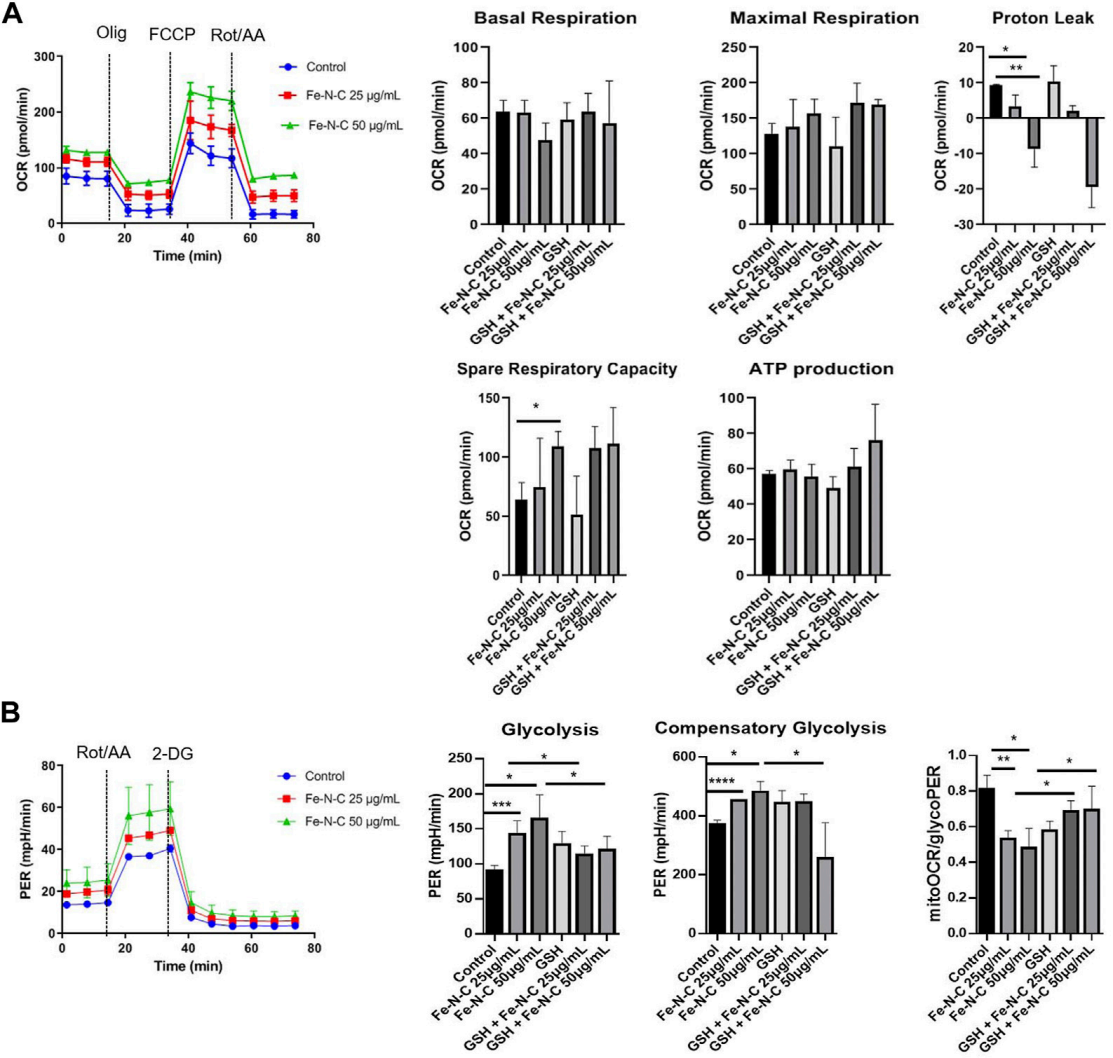
The oxygen consumption rate (OCR) and proton efflux rate (PER) influenced by Fe-N-C in breast cancer cells. MDA-MB-231 cells were seeded in XF 96 cell-culture plates and incubated with Fe-N-C and GSH (4 mM) for 4 h. OCR **(A)** and PER **(B)** were monitored using the Seahorse XFe96 extracellular flux analyzer in real time. Mitochondrial respiration was indicated by stimulation with oligomycin (Olig), carbonyl cyanide p-trifluoromethoxyphenylhydrazone (FCCP), rotenone (Rot) and antimycin A (AA). Glycolysis was stimulated by rotenone (Rot), antimycin A (AA) and 2-deoxyglucose (2-DG) in MDA-MB-231 cells. Data are representative of three independent experiments. Summary data are shown as the mean ± S.E.M. with *p* values determined by two-tailed Student’s *t*-test (*p < 0.05, **p < 0.01, ***p < 0.001).

Sine TNBC have an elevated glycolytic and concomitantly lower OXPHOS (Pelicano et al., 2014; Urra et al., 2018; Wu et al., 2020). We speculated that Fe-N-C SAzymes can induce the metabolic alterations, causing mitochondria repurpose from ATP synthesis to ROS production (Mills et al., 2016). Reactive oxygen species are mainly generated in mitochondria and massively produced while mitochondrial dysfunction occurs, concurrently with attenuation of mitochondrial OXPHOS and glycolysis. To determine the level of tumor mitochondrial energy metabolism after exposure to Fe-N-C, a Seahorse XF^e^96 extracellular flux analyzer was used to measure oxygen consumption rate (OCR) and proton efflux rate (PER) in real time. OCR is employed to study mitochondrial OXPHOS function while PER represents glycolytic metabolism. From OCR analysis, Fe-N-C SAzymes slightly decreased the basal respiration but did not show statistical significance. While the maximal respiration and ATP production did not show any change, spare respiratory capacity increased after Fe-N-C 50 μg/ml treatment. Consistent with the results of basal respiration, Fe-N-C reduced proton leak, which was independent on the •OH generation because there was no reverse after GSH treatment (Figure 7A). On the contrary, glycolysis elevated after Fe-N-C treatment compared with the control. Basal glycolysis and compensatory glycolysis represented by rotenone and antimycin A stimulation increased and attenuated when •OH radicals were blocked. Based on PER analysis, Fe-N-C reduced mitoOCR/glycoPER, which was consistent with the result of elevated basal glycolysis and compensatory glycolysis (Figure 7B). Taken together, Fe-N-C significantly upregulated glycolysis in MDA-MB-231 which was blocked by GSH, while causing minor changes to OXPHOS. In addition, OCR and ECAR in 4T1 cells were measured by seahorse XF^e^96 extracellular flux analyzer and the consistent conclusions were obtained (Supplementary Figure S6).

### CDT/PTT of Fe-N-C SAzymes *in vivo*

**FIGURE 8:**
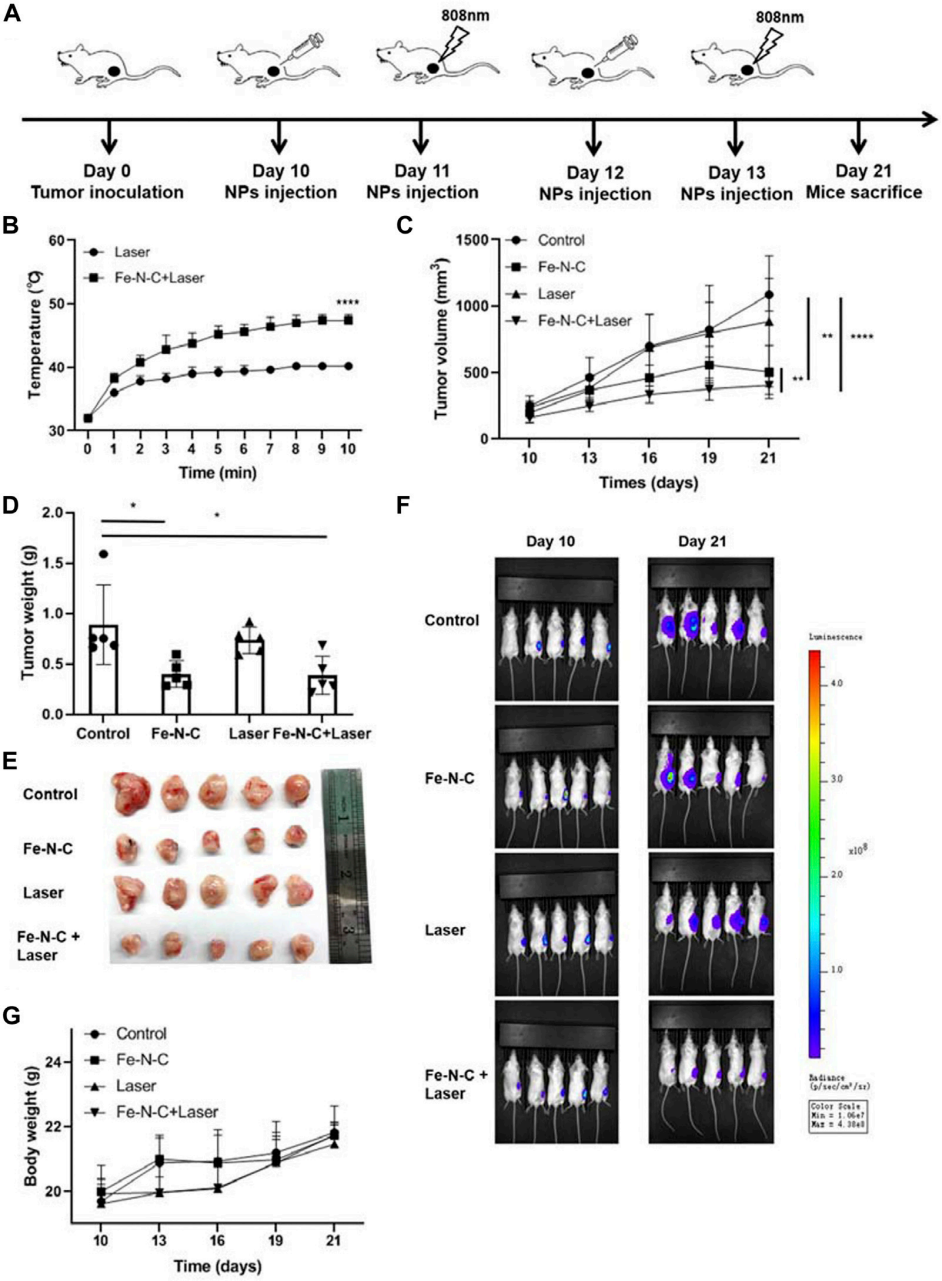
CDT/PTT effect of Fe-N-C SAzymes *in vivo*. **(A)** Schematic diagram of our experiments to study the antitumor effect of Fe-N-C SAzymes *in vivo*. Subcutaneous tumor was established by luciferase-labeled 4T1 cells. Fe-N-C treatment was performed by intratumoral injection. **(B)** The temperature changes of tumor surface were recorded during 808 nm laser exposure (1.0 W cm^−2^) for 10 min, 24 h after Fe-N-C (200 μg) intratumoral injection. **(C)** Tumor volumes were determined and the tumor growth curves were plotted for indicated treatments (mean ± S.E.M.). **(D)** and **(E)** Tumor weight of sacrificed mice at day 21 (mean ± S.E.M.). **(F)** *In vivo* fluorescence images of tumor-bearing living mice before and after treatment. **(G)** Body weight changes were recorded during treatments. *In vivo* data are representative of two independent experiments. Summary data are shown as the mean ± S.E.M. with *p* values determined by two-tailed Student’s *t*-test (*p < 0.05, **p < 0.01, ***p < 0.001, ****p < 0.0001).

We next investigated the effect of Fe-N-C SAzymes on the ability of 4T1 mammary carcinoma bearing B6 mice. As shown in Figure 8A, an 808 nm laser was arranged 24 h after Fe-N-C SAzymesr intratumoral injection. Tumor temperatures were recorded every 1 min for 10 min. During phototherapeutic process, the temperature of Fe-N-C treated tumors increased significantly from 32°C to 46°C (Figure 8B). During 12 days, the tumor volume, tumor weight and fluorescence value were significantly inhibited by Fe-N-C SAzymes and further suppressed when plus 808 nm laser irradiation (Figures 8C–F). Fe-N-C medicated phototherapy achieved exceptional antitumor efficacy with >50% tumor growth inhibition. It was noted that there was no significant weight loss among all groups representing unobvious adverse effect (Figure 8G) which is consistent with *in vitro* cytotoxicity experiment that cytotoxicity was evaluated in breast cancer cell line MDA-MB-231 than normal breast cell line MCF-10A (Supplementary Figure S5). Under these experimental conditions, synergistic cancer therapy of chemodynamic therapy (CDT) and photothermal therapy resulted in the significant inhibition of tumor growth.

### Fe-N-C SAzymes breaks innate immune tolerance

**FIGURE 9:**
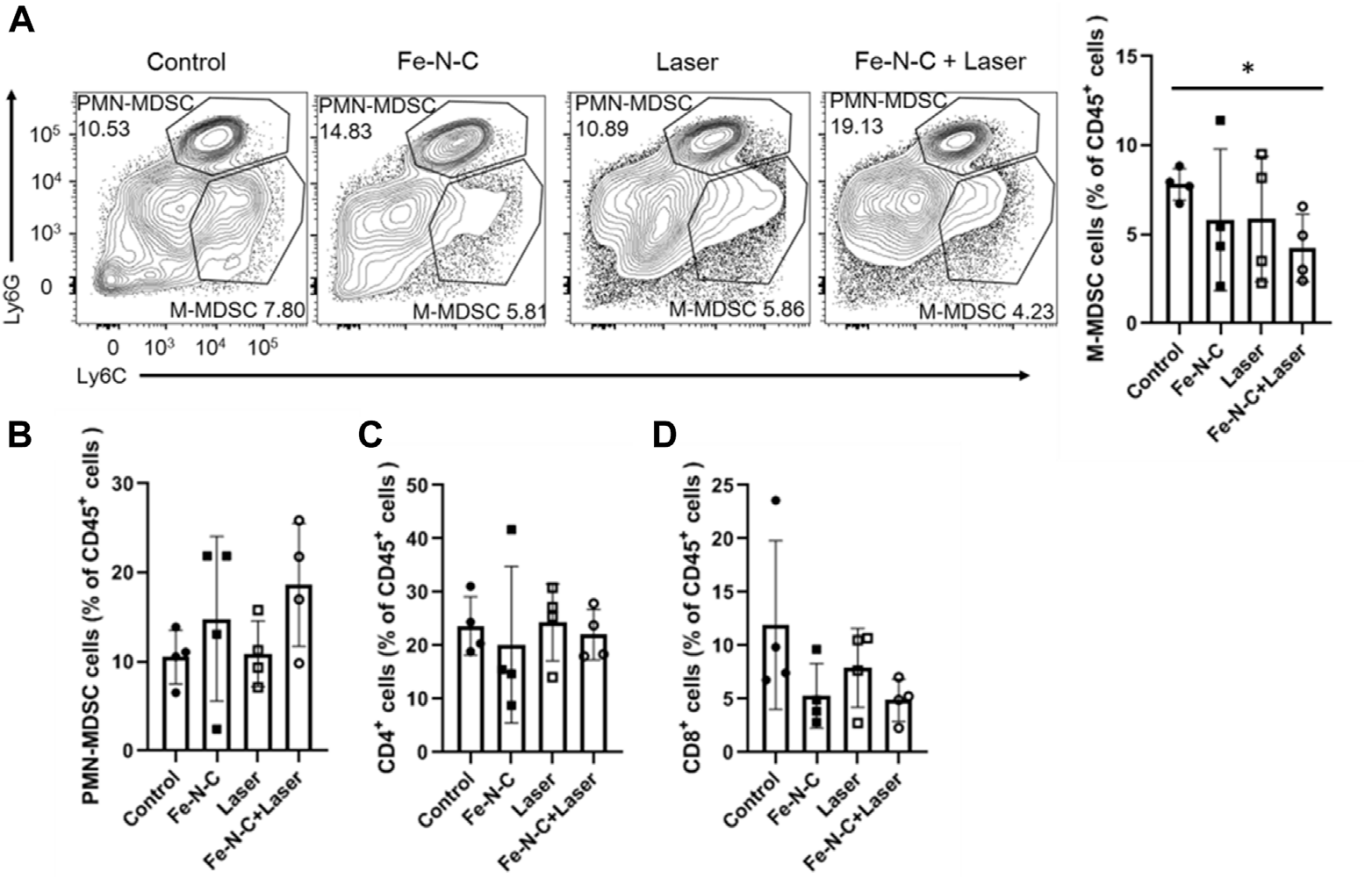
Fe-N-C SAzymes breaks innate immune tolerance. **(A)** Flow cytometry analysis of the percentage of M-MDSC cells expressing CD11b+, Ly6C + and Ly6G-markers. **(B)** Flow cytometry analysis of the percentage of PMN-MDSC cells expressing CD11b+, Ly6C- and Ly6G + markers. **(C)** Flow cytometry analysis of the percentage of CD4^+^ cells expressing CD11b- and CD4^+^ markers. **(D)** Flow cytometry analysis of the percentage of CD8^+^ cells expressing CD11b- and CD8α+ markers. Data are representative of two independent experiments. Summary data are shown as the mean ± S.E.M. with *p* values determined by two-tailed Student’s *t*-test (*p < 0.05, **p < 0.01, ***p < 0.001, ****p < 0.0001).

In addition to inducing tumor cells apoptosis, CDT/PTT also brings potent antitumor immune responses by the generation of different cytokines, exodus of post-apoptotic components and heat shock proteins (HSPs). Those tumor-associated antigens released help to reshape the immune microenvironment (Joffre et al., 2009). Local PTT increases the local temperature of the targeted tumor and activates immunological system called *in situ* photoimmunotherapy (ISPI), which was demonstrated successfully in patients with advanced melanoma (Li et al., 2010). To examine the role of Fe-N-C SAzymes in regulating tumor infiltrating immune cell responses, we further analyzed four groups of mice with subcutaneous breast cancer. Compared to control, monocytic-myeloid derived suppressor cells (M-MDSC) significantly decreased after Fe-N-C SAzymes treatment combined with laser illumination (Figure 9A). On the other hand, tumor infiltrating polymorphonuclear-myeloid derived suppressor cells (PMN-MDSC), CD4^+^ T cells and CD8^+^ T cells did not show statistically significant changes (Figures 9B–D). M-MDSC and PMN-MDSC which belong to the innate immune system are the main members of tumor immune tolerance, while CD4^+^ T cells and CD8^+^ T cells play an important role in antitumor immunity. These findings suggest that our Fe-N-C SAzymes reprogrammed the tumor immune landscape by breaking innate immune tolerance *via* suppressing tumor infiltrating M-MDSC.

## Discussion

Besides chemotherapy and radiotherapy, CDT/PTT is a new therapeutic method to generate ROS or convert light energy into hyperthermia to inhibit cancer cell proliferation (Lv et al., 2021). Fe-N-C SAzymes treatment plus laser exposure resulted in a more than 90% decrease in tumor cell viability, better than laser exposure alone or Fe-N-C SAzymes treatment alone. Annexin V/7-AAD assay and cleaved-caspase three protein expressions detected by Western-Blot revealed that many apoptotic cells were observed in the tumor treated with Fe-N-C SAzymes plus laser exposure. For ROS-related ferroptosis is well known in regulating cell death, it is characterized by iron-dependent accumulation of lipid hydroperoxides (Yang and Stockwell, 2016; Chang et al., 2021). Ferroptosis was also detected after Fe-N-C treatment, but the mRNA of the main ferroptosis related genes did not show any change (Supplementary Figure S3A) as well as the GPX4 protein expression, which is always down-regulated in ferroptosis (Supplementary Figure S3B). Besides, there was no change in cell proliferation after ferrostatin-1 blocked which was known as ferroptosis inhibitor (Supplementary Figure S3C). Together, these results suggested that apoptosis rather than ferroptosis was the main form of antitumor process in Fe-N-C SAzymes mediated CDT/PTT.

Nevertheless, chest metastasis is common but difficult to handle in TNBC. Xenograft subcutaneous tumor shrinking suggests that the dual CDT/PTT therapeutic methods revealed a prospective application. Some other nanozymes have been reported for effective cancer therapy, among which CDT and PTT play a key role. For example, PCN-224-Pt by decorating Pt nanozymes on photosensitizer integrated MOFs, zirconium-ferriporphyrin MOF (Zr–FeP MOF) nanoshuttle (Chen et al., 2017) and our group studied nanosystems (Wei et al., 2013; Odda et al., 2019; Odda et al., 2020; Sahar et al., 2020). The common antitumor mechanism is that nanozymes are able to utilize H_2_O_2_ to produce abundant reactive oxygen species including hydroxyl radical (•OH) and/or singlet oxygen (^1^O_2_) to fight cancer (Wu et al., 2021). Our nanoagent exhibits an effective ROS conversion and a safety temperature rise.

ROS generation is always produced in the mitochondria, which is also the main organelle for ATP production in TNBC (Richards et al., 2018). Therefore, modulation of energy metabolism in cancer cells to inhibit ATP production and steer ROS generation, is now considered as a novel strategy for treatment of metastatic cancer. Fe-N-C SAzymes caused minor changes to OXPHOS for there was no effect on basal respiration both in MDA-MB-231 and 4T1 but different effect on maximum respiration and proton leak. The difference between the 2 cell lines may be caused by cell number or FCCP sensitivity and so on. However, ATP production was not affected by Fe-N-C SAzymes, which mean both ATP synthesis and ROS production exist in mitochondria. Fe-N-C significantly upregulated glycolysis both in MDA-MB-231 and 4T1, which was blocked by GSH in MDA-MB-231 cell line. Both CDT and PTT could activate the immune system and recruit different immune cells into the tumors, thus exerting a strong antitumor effect. In our experiments, Fe-N-C SAzymes modulated tumor infiltration of immune cells. M-MDSCs, as a group of immunosuppressive cells, express myeloid lineage marker CD11b, induce T cells dysfunction and further promote tumor cell proliferation (Chen et al., 2019). Our results exhibited that PTT based on Fe-N-C SAzymes could enhance immunotherapy of TNBC by suppressing M-MDSC. However, how did enhanced glycolysis modulate the immune suppressive activity of MDSCs needs further study. Overall, this study provides a promising strategy to develop other artificial nanozymes for effective combination therapy of cancer.

## Conclusion

In conclusion, we have successfully synthesized Fe-N-C single atom nanozymes by a simple method. The as-made nanoagent offers a new and promising strategy to attain enhanced CDT/PTT for TNBC and achieve high therapeutic efficacy against cancer cells *in vivo* and *in vitro*. The results confirmed that Fe-N-C SAzymes could impair mitochondrial OXPHOS, promote glycolysis and generate ROS by decomposing H_2_O_2_ into •OH, eventually induce tumor cells apoptosis. It was noted that under 808 nm irradiation, Fe-N-C nanoagent displayed enhanced cell cytotoxicity and inhibition of cell proliferation by suppressing tumor infiltrating M-MDSC. Chemodynamic and photothermal therapy based on multifunctional nanoagents might provide a new opportunity for clinical treatment of TNBC chest metastasis. Overall, this work provides a new route to the use of a single nanoagent for multifunctional therapy.

## Data Availability

The raw data supporting the conclusion of this article will be made available by the authors, without undue reservation.
