# Supplementary material for: The single-atom iron nanozyme mimicking peroxidase remodels energy metabolism and tumor immune landscape for synergistic chemodynamic therapy and photothermal therapy of triple-negative breast cancer
Source: Front Bioeng Biotechnol. 2022 Oct 26;10:1026761. doi: 10.3389/fbioe.2022.1026761 (PMC9644204; doi:10.3389/fbioe.2022.1026761)
Supplement: Supplementary file 1 [file DataSheet1.docx]

**The single-atom iron nanozyme mimicking peroxidase** **remodels energy metabolism and tumor immune landscape for** **synergistic chemodynamic therapy and photothermal** **therapy of triple-negative breast cancer**

**Xiaojun Qian^1,*^, Ronghua Shi^2,*^, Jian Chen^1^, Yong Wang^1^, Xinghua Han^1^, Yubei Sun^1^, Cong Ling^3^, Gang Wang^1^, An-Wu Xu^1^, and Yueyin Pan^1^**

**Contents:**

⚫ **Figure S1.** XRD pattern of Fe-N-C SAzymes.

⚫ **Figure S2.** UV-Vis-NIR absorption spectrum of Fe-N-C SAzymes (500 μg/mL).

⚫ **Figure S3.**  Ferroptosis independent manner of Fe-N-C induced cell death.

⚫ **Figure S4.**  Cell viability of Fe-N-C SAzymes in MCF-10A and MDA-MB-231 cells.

⚫ **Figure S5.**  Intracellular H2O2 detection in MCF-10A and MDA-MB-231 cells.

⚫ **Figure S6.**  The oxygen consumption rate (OCR) and Extracellular Acidification (ECAR) influenced by Fe-N-C in4T1.


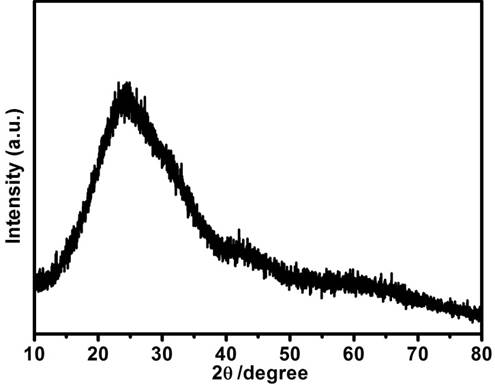


**Figure S1.** XRD pattern of Fe-N-C SAZymes.


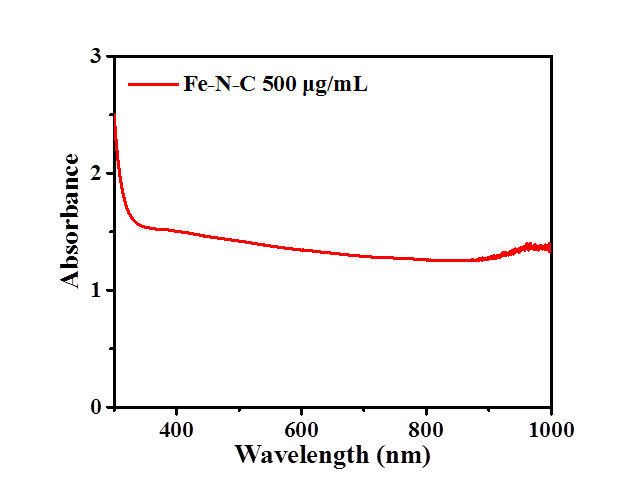


**Figure S2.** UV-Vis-NIR absorption spectrum of Fe-N-C SAzymes (500 μg/mL).


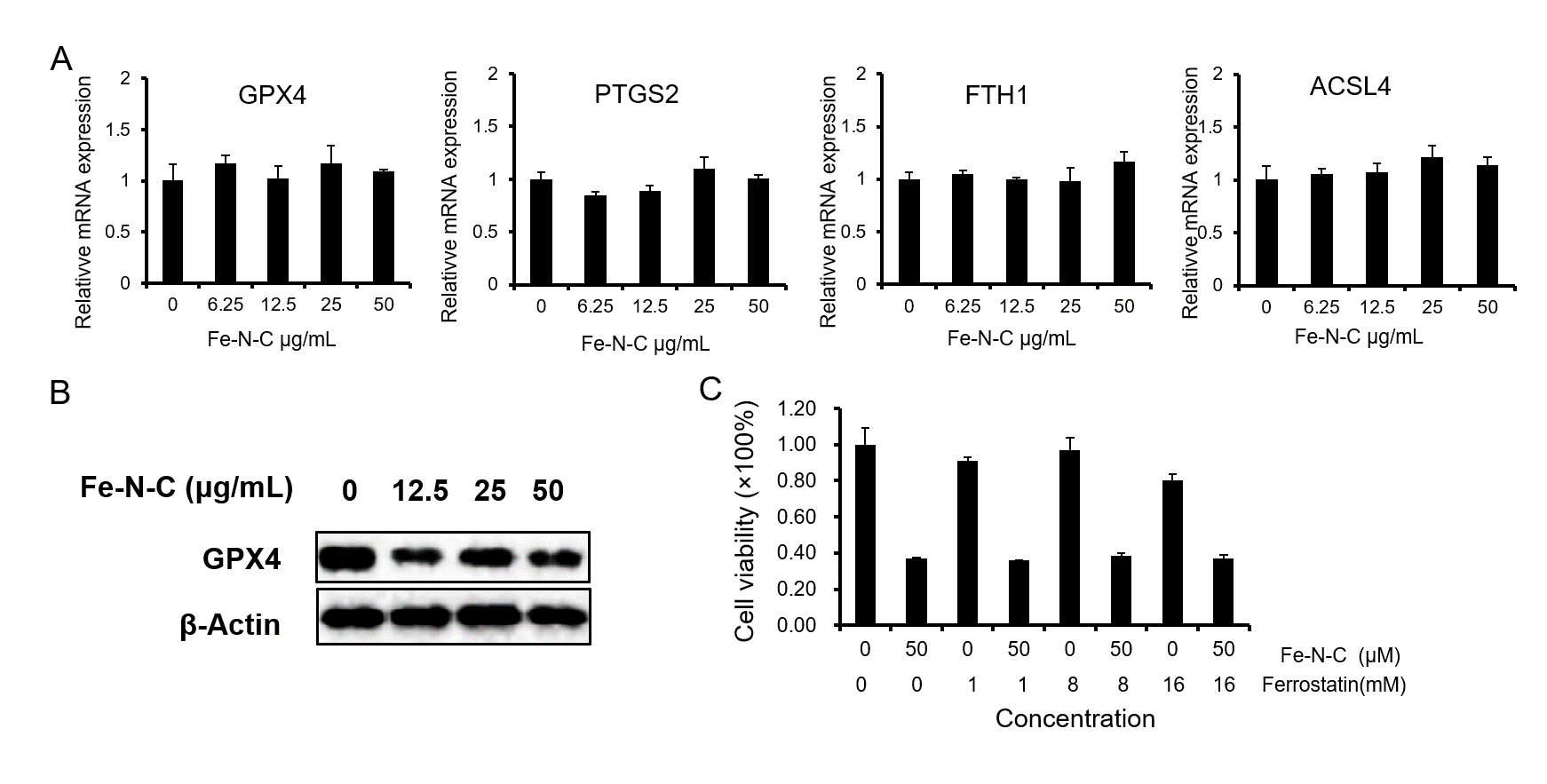


**Figure S3.** Ferroptosis independent manner of Fe-N-C induced cell death. (A) RT-qRCR analysis of the mRNA expression of GPX4, PTGS2, FTH1 and ACSL4 after Fe-N-C treatment for 4 h in MDA-MB-231 cells. (B) Immunoblot analysis of GPX4 protein expression after Fe-N-C treatment for 4 h in MDA-MB-231 cells. (C) Ferrostatin-1 was added at indicated concentration combined with or without Fe-N-C SAzymes for 4 h. Cell viability of MDA-MB-231 cells was detected by CCK-8 assay. Data are representative of two (B) or three (A, C) independent experiments.


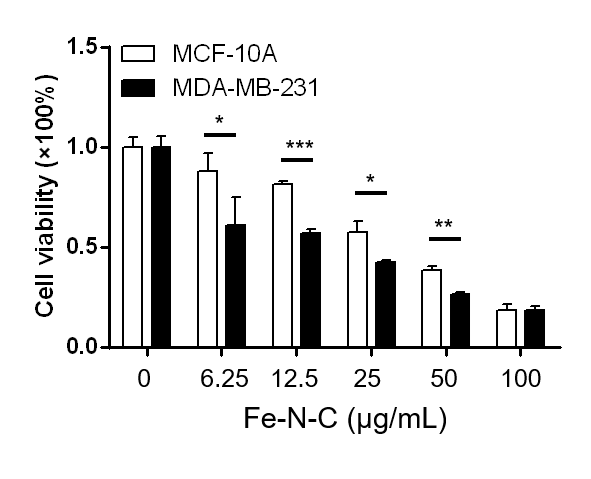


**Figure S4.** MCF-10A and MDA-MB-231 cells were treated by Fe-N-C with different concentrations for 4 h. The cell viability was measured by CCK8 assay.


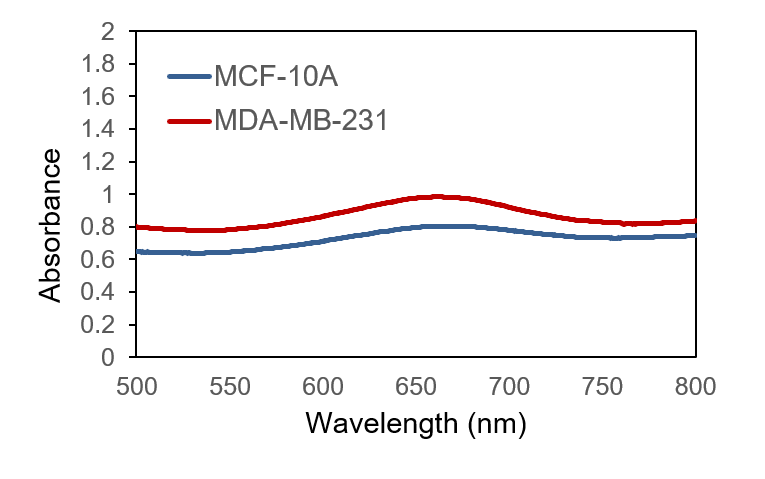


**Figure S5.** Intracellular H_2_O_2_ detection through colorimetric assay of TMB as substrate by UV-Vis-NIR absorption spectra at 652 nm in MCF-10A and MDA-MB-231 cells. Each group contained 7$\times$10^5^ cells and Fe-N-C 25 μg/mL.


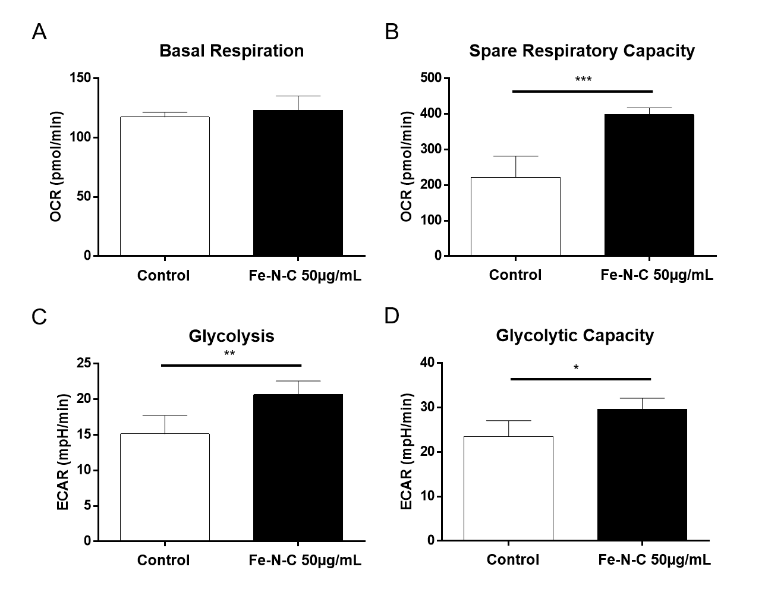


**Figure S6** The oxygen consumption rate (OCR) and Extracellular Acidification (ECAR) influenced by Fe-N-C in 4T1 cells. Cells were seeded in XF 96 cell-culture plates and incubated with Fe-N-C for 4 h. OCR (A, B) and ECAR (C, D) were monitored using the Seahorse XF^e^96 extracellular flux analyzer in real time. Mitochondrial respiration was indicated by stimulation with oligomycin (Olig), carbonyl cyanide p-trifluoromethoxyphenylhydrazone (FCCP), rotenone (Rot) and antimycin A (AA). Glycolysis stress test was stimulated by glucose, oligomycin (Olig) and 2-deoxyglucose (2-DG) in 4T1 cells. Data are shown as the mean ± S.E.M. with *P* values determined by two-tailed Student’s t-test (**P* < 0.05, ***P* < 0.01, ****P* < 0.001).
